# Vinculin and the mechanical response of adherent fibroblasts to matrix deformation

**DOI:** 10.1038/s41598-018-36272-9

**Published:** 2018-12-19

**Authors:** Kathryn A. Rosowski, Rostislav Boltyanskiy, Yingjie Xiang, Koen Van den Dries, Martin A. Schwartz, Eric R. Dufresne

**Affiliations:** 1 Department of Materials, ETH Zürich, 8093 Zürich, Switzerland; 2 Department of Mechanical Engineering and Materials Science, Yale University, New Haven, CT 06511 USA; 3 Cardiovascular Research Center and Department of Medicine (Cardiology), Yale University School of Medicine, New Haven, CT 06511 USA; 4 Departments of Cell Biology and Biomedical Engineering, Yale University, New Haven, CT 06511 USA; 5 Present Address: Department of Cell Biology, Radboud Institute for Molecular Life Sciences, Radboud University Medical Center, Nijmegen, The Netherlands

## Abstract

Cells respond to the mechanics of their environment. Mechanical cues include extracellular matrix (ECM) stiffness and deformation, which are primarily sensed through integrin-mediated adhesions. We investigated the impact of ECM deformation on cellular forces, measuring the time-evolution of traction forces of isolated mouse fibroblasts in response to stretch and release. Stretch triggered a marked increase of traction stresses and apparent stiffness. Expression of the focal adhesion protein vinculin not only increased baseline traction forces, but also increased dissipation of mechanical energy, which was correlated with the cells’ failure to recover baseline traction forces after release of stretch.

## Introduction

Cells in the body are continually strained through the action of their neighbors and the surrounding extracellular matrix (ECM)^1^. In order to maintain their structure and function, these cells sense and respond to deformation^2^. *In vitro*, non-muscle cells respond to sustained mechanical changes through alterations of cytoskeletal components, such as the formation of new actin stress fibers^3^ or their re-alignment^4–6^. On shorter time scales, cells have been observed to stiffen when strain is applied to adhesions^7^ or the whole cell^8^. Stiffness is also strongly influenced by contractile machinery, such as acto-myosin^7,9–11^, and is correlated with higher traction forces^10^.

Cell stiffness is typically probed with the tip an atomic force microscope (AFM) or an adhered magnetic bead which is pulled (magnetic tweezers) or twisted (magnetic twisting cytometry, MTC). These approaches probe local material properties and are primarily sensitive to the stiffness of the cortex. On the other hand, mechanics at the whole-cell level can be assessed using traction force microscopy (TFM), which quantifies spatially-resolved stresses exerted by a cell on the underlying matrix^12^. In contrast to AFM and MTC, it measures forces spontaneously generated by cells, rather than the mechanical resistance to externally applied forces.

Deformation of the ECM drives a mechanical response on the whole-cell scale. Long-term repeated stretch leads to cytoskeletal reorganization and, in many cases, a lowering of cell traction forces^13,14^. During a single stretch however, traction forces increase^3,15–17^. When stretch is sustained, a slow drop in force, reminiscent of the response of a viscoelastic material, has been reported^16^. At the release of stretch, traction forces were observed to initially drop before slowly returning to baseline^15,18^. Because this drop in force after a stretch-release cycle corresponds to a decrease in stiffness (measured through MTC)^19,20^, it has been suggested that the cytoskeleton fluidizes in response to stretch and release, and then re-solidifies to bring the cell back to its original state^18,19,21^. Measurements of cells stretched between parallel plates suggest that cells behave like a heterogeneous viscoelastic material and thus cannot be described by simple models^22^.

Here, we trace the time-evolution of traction forces throughout sustained stretch and release of the ECM, quantifying cellular response to deformation over timescales from a few seconds to hundreds of seconds. While the long-term response to stretch is variable, all cells displayed a sharp increase in force at stretch and a sharp decrease in force at release of stretch. The magnitudes of these force changes are linearly related to the applied strain, allowing us to define an apparent whole-cell stiffness, which includes potential contributions from both passive material stiffness and active contractility. The apparent stiffness is proportional to the traction forces, and shows a marked increase in the stretched state. The focal adhesion protein vinculin^23–27^ is found not only to increase baseline stresses, as previously reported, but also to enhance dissipation of mechanical energy throughout the stretch-and-release process. In both cell types, mechanical dissipation during the stretch and release cycle is correlated with an inability to re-establish baseline traction forces after the cycle.

## Materials and Methods

### Cell culture

Vinculin KO (Vin^−/−^) mouse embryonic fibroblasts (MEFs) and control cells reconstituted with EOS-vinculin (Vin^−/−^; Vin-EOS^+^) were grown in DMEM with 10% FBS, 1% L-Glutamine and 1% Pen-Strep (Invitrogen). Cells were maintained at 37 °C, 5% CO_2_ and passaged upon confluency. 1–2 days before each experiment, TFM substrates were coated with fibronectin (20 µg/ml, Sigma-Aldrich) and single cells were plated on top, where they adhered and spread. The day of the experiment, cells were stained with Cell Tracker Dye (Molecular Probes) and transferred to medium with 10 mM HEPES. TFM experiments were done at 37 °C, in a lab-made temperature chamber mounted on the microscope.

### Substrate preparation

TFM substrates were prepared by coating glass-bottom dishes (Willco Wells) with a 750–950 µm thick layer of silicone gel (Dow Corning Toray, a 1:1 mixture of components CY 52–276-A and CY 52–276-B). The silicone was cured at room temperature overnight to obtain a gel with a Young modulus of 3 kPa^12^. Fluorescent beads (500 nm radius, Invitrogen) were adsorbed onto the surface of the silicone, in a solution of borate buffer (components from Sigma-Aldrich) and EDC (Sigma-Aldrich). Before the cells were attached, substrates were UV-sterilized for 20 minutes.

### Stretching set up

An indenter was constructed with a translation stage from Thorlabs (Z812B), and glass capillary tubes (2.0/1.12 mm outer/inner diameter, World Precision Instruments, Inc). Upon indentation, the silicone in the middle of the tube was pushed down and the surface stretched^18^. The resulting strain at the center of the tube was nearly equi-biaxial and its magnitude increased with the indentation depth. In these experiments, the indentation depth varied from 200 to 600 µm. The capillary tube height was controlled through the APT program (Thorlabs), and moved at a constant speed of 0.3 mm/sec during both indentation and release. Resulting substrate deformation profiles are displayed and quantified in Supplementary Fig. S2.

### Traction force microscopy

The cells adhered well to the fibronectin, forming adhesions and pulling on the TFM substrate. After each time-course, cells were lifted from the surface with trypsin (0.5% with EDTA, Invitrogen) to acquire a zero-stress reference state. Beads were imaged throughout the experiment on a spinning-disk confocal microscope (Andor Revolution, on a Nikon Ti Eclipse) at 40x/0.60 NA. The applied strain at each time point was measured by the determination of the far-field deformation. A translation and affine transformation was fit to the far-field bead displacements relative to the reference image in MATLAB (MathWorks, Natick, MA), by solving a non-linear least squares problem. Once these deformations were subtracted from the images, displacements due to the cell were tracked^28^ and TFM analysis was performed as previously described^12^. Rheology measurement of the substrates showed that the silicone gel was linear in the range of applied strains, and so calculations in the stretched state took into account only the decreased thickness of the substrate due to indentation. From the integration of traction stresses over space, forces and total force magnitudes were calculated. Errors in the total force measurements due to errors in removing the affine deformation are quantified in the Supplementary Fig. S2.

### Immunofluorescence

Cells were plated on glass coverslips coated with silicone and fibronectin, as above. After 2–3 days in culture, they were fixed in 3.7% formaldehyde for 10 minutes at room temperature (RT). They were blocked for >30 minutes in a solution containing 1X PBS, fish gelatin, normal goat serum, normal donkey serum, bovine albumin serum and 0.25% Triton X-100. After blocking, cells were incubated in primary antibodies diluted in block for 1.5 hrs at RT: paxillin (rabbit) @ 1:250, vinculin (mouse) @ 1:500, pMLC (mouse) @ 1:200. Secondary antibodies were diluted in block and incubated for 45 min at RT: Alexa 532 anti-rabbit @ 1:250, Cy5 anti-mouse @ 1:250. Phalloidin-Alexa594 (Invitrogen) was diluted to 1:40 in PBS and incubated 20 min at RT. Stained cells were then mounted in Prolong Gold containing DAPI. Phalloidin images were taken on a Zeiss LSM 510 confocal at 100x/1.4 NA. All other fluorescent images were taken on a Zeiss Axioimager M1 at 63x.

### Cell length measurements

Due to the spindle-like morphology of the cells, and the thick silicone layer underneath them, measurements of cell length from fluorescent imaging were ambiguous. On the other hand, strain energy^29^ was consistently localized to two clear maxima on either end of the cell. The distance between the centroids of the strain-energy peaks is used here as a measure of cell length.

### Analysis and Statistics

Traction forces were calculated for cells which remained well-adhered throughout the entire stretch and release protocol. The quality of the TFM measurements was assessed using the vector sum of the traction stresses, which should be zero. We discarded any cells with more than a 15% excess of the vector sum compared to the scalar sum, $|\int dA({\sigma }_{xz}\hat{x}+{\sigma }_{yz}\hat{y})|\ge 0.15\int dA|{\sigma }_{xz}\hat{x}+{\sigma }_{yz}\hat{y}|$. This has been used previously as a classic TFM quality measurement^30^.

Analysis and statistics of the differences between different cell responses was done using MATLAB. To determine significance between the two sample populations of different sizes, an unequal variances (Welch’s) t-test was performed. Significance between different time points of the same sample population was determined using the two-tailed paired Student’s t-test. When distributions were not clearly normal, the Kolmogorov-Smirnov test was used for a second assessment of significance.

To measure correlation, Pearson’s correlation coefficient, *r*, was calculated between two experimentally independent variables. For fit lines, the coefficient of determination, *R*^2^, was used to measure the goodness of fit through linear regression.

## Results and Discussion

### Vinculin Knock-Out cells exert lower baseline traction forces

**Figure 1:**
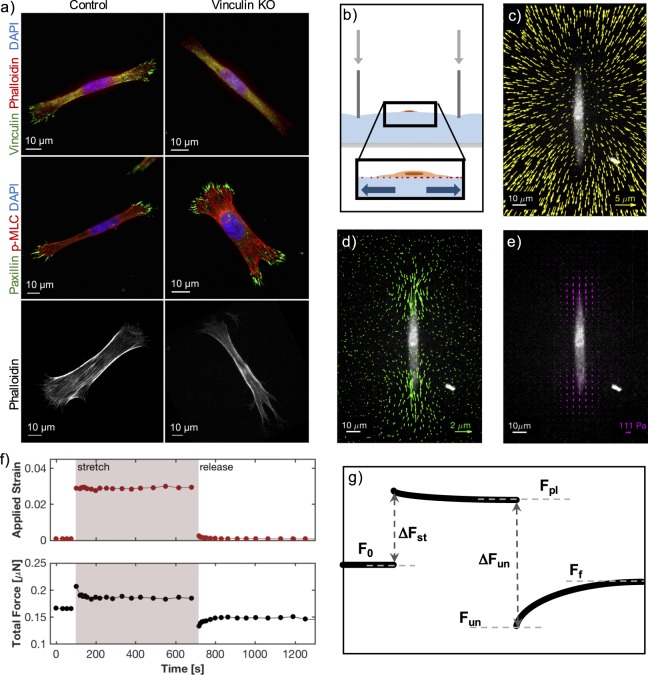
Experimental design. (**a**) Immunofluorescent images of adherent control (left panels) and vinculin KO (right panels) cells on silicone-coated glass coverslips. Vinculin and paxillin are focal adhesion proteins. Phalloidin stains for actin and DAPI for cell nuclei. p-MLC (phospho-myosin light chain) marks active myosin. (**b**) Schematic of the set-up for cell stretching. As a thick silicone substrate is indented with a glass capillary tube, the surface is stretched equi-biaxially, stretching the attached cell. (**c**) Beads embedded under the silicone surface are tracked from an un-stretched to stretched state, showing the magnitude of applied strain. (**d**) The far-field strain is subtracted from the displacement map of the embedded beads (in c), leaving only the residual displacements caused by the pulling of the cell. (**e**) Stresses are calculated from the displacement map (in d). (**c**–**e**) White lines represent the scale bar of the fluorescent cell image. Colored arrows represent the scale bar of the overlaid displacements or stresses. (**f**) An example of real data showing the evolution of force changes. The applied strain is plotted over the time of the experiment, showing the pre-stretched, stretched and post-release state of the cell (above). The force magnitudes applied by the cell are integrated and shown throughout the same course of the experiment (below). (**g**) A schematic showing the force trace over time and the quantities focused on in our analysis.

We investigated the stretch response of individual mouse embryonic fibroblasts under the conditions of low cell density (*i.e*. without close neighbors). We considered two cell populations: vinculin knock-out (KO) cells and control cells, in which vinculin was re-introduced. The response of a cell to stretch likely involves mechanosensitive integrin adhesions, of which vinculin is an important component. Vinculin plays a role in force transduction across the focal adhesion^31,32^, contributes to traction force generation^23,24,33,34^, and is responsive to force itself^35,36^. Thus, we expected loss of this protein to affect a cell’s mechanical response. Vinculin, however, is not integral to the basic function of focal adhesions. In the absence of vinculin, cells still adhered to the substrate and generated traction stresses. Immunofluorescent staining of the cells confirmed the absence of vinculin in our KO cells (Fig. 1a top, Fig. S1a). However, the focal adhesion protein paxillin was still strongly expressed in the adhesions (Fig. 1a, middle). In addition, the morphology of vinculin KO cells was not dramatically affected and phalloidin staining showed that actin stress fibers continued to traverse the length of the cell (Fig. 1a, bottom).

Both cell types were plated on 3 kPa silicone TFM substrates coated with fibronectin. Standard traction force microscopy was performed to achieve a basal level of traction forces on all the cells. Consistent with previous reports^23,24,32–34^, we found that baseline traction forces were decreased by around 35% in vinculin KO cells relative to control (0.20 µN for control cells and 0.13 µN for vinculin KO cells; p = 0.006, Fig. S1b). It has been previously shown^29,37^ that cellular forces can be dependent on cell size. Here, we did not find any correlation between size and baseline traction forces (r = −0.25 for control cells, r = −0.16 for vinculin KO cells; Fig. S1c), nor did we see any significant difference in the length distributions of the two populations, which averaged around 60 µm long (mean of 59.9 µm for control cells, mean of 54.9 µm for vinculin KO cells, p = 0.17; Fig. S1d).

### Fibroblasts increase traction forces during stretch

After recording the baseline traction stresses, we stretched the substrate in the vicinity of the cell by indentation with a thin glass tube^18^. The substrate directly below the indenter wall was compressed, but the surface under the hollow center of the tube was stretched biaxially (Figs 1b, S2a). While the substrate surface was curved on the scale of the tube diameter, it was essentially flat on the scale of the cell (Fig. S2b,c). By coordinating the motion of the indenter and microscope objective, we were able to refocus the microscope on the cell within seconds of indentation. Applied strains were determined by analyzing displacements of beads far away from the cell (Figs 1c, S2d). We subtracted this far-field deformation to reveal the cell’s contribution to the substrate displacement (Fig. 1d). These residual displacement fields were analyzed to determine the cellular traction stresses (Fig. 1e).

We studied the response of cells to subsequent stretch and release (Fig. 1f). To facilitate comparison of traction forces over time, we reduced each traction map to a single force value by integrating the magnitude of the traction stresses over the whole cell^29,30,38^. The total traction force throughout the stretch and release procedure for a typical experiment is shown in Fig. 1f and schematized in Fig. 1g. Before stretch, cells maintained a stable traction force, *F*_0_. Immediately after stretch, all cells increased their traction forces by an amount, ${\rm{\Delta }}{F}_{st}$. Stretch was held for about ten minutes, during which time cellular forces typically established a plateau value, *F*_*pl*_. Immediately after release of stretch (“un-stretch”), all cells showed a sharp decrease in their traction forces, ${\rm{\Delta }}{F}_{un}$, reaching a minimum, *F*_*un*_. Traction forces typically climbed back toward a final resting value ten minutes later, *F*_*f*_.

### Cells have a linear mechanical response to stretch over short times

**Figure 2:**
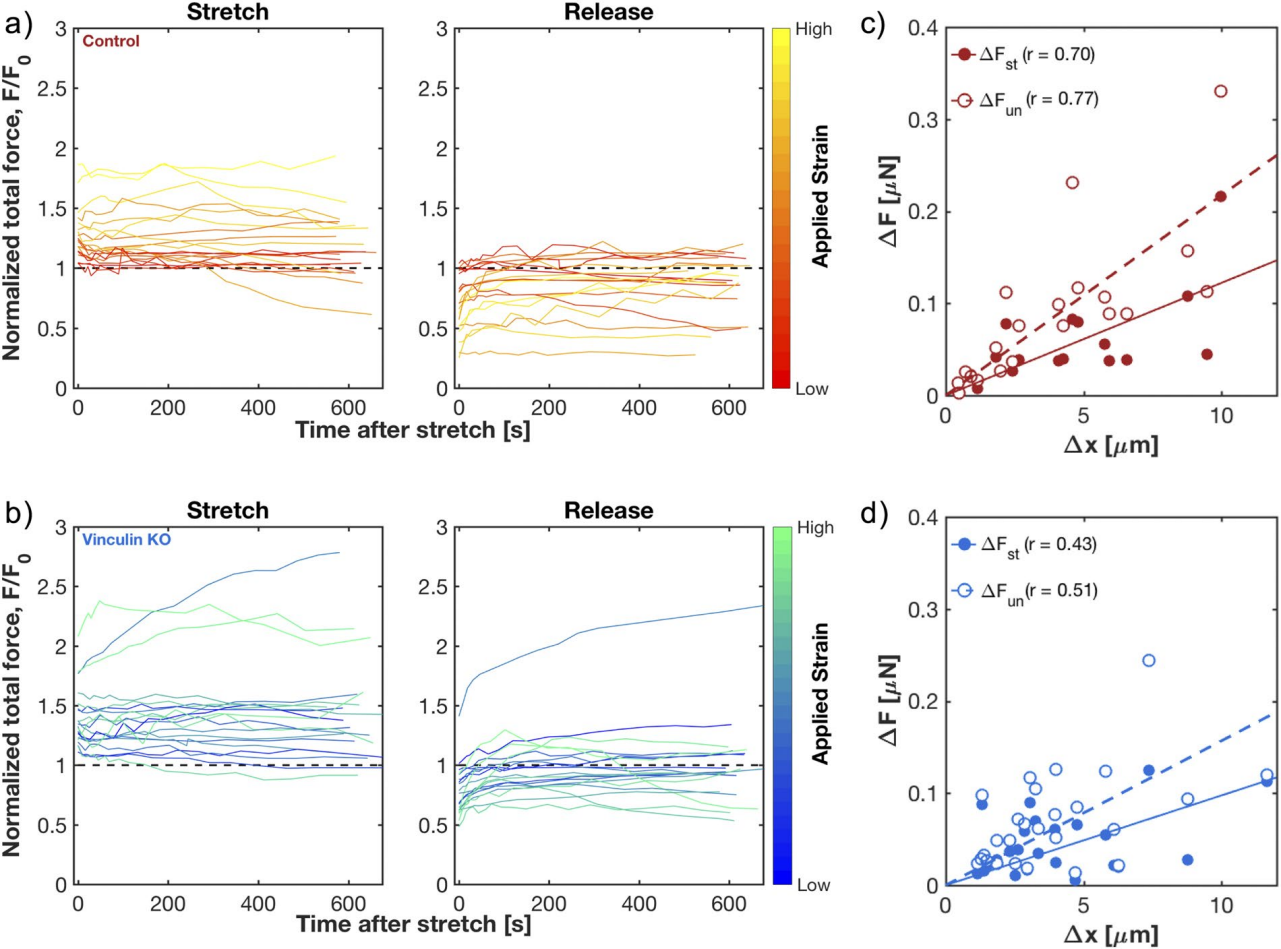
Mechanical response of fibroblasts to stretch. (**a**,**b**) Traces of individual control cells (**a**) and vinculin KO cells (**b**), showing force changes over the time of stretch (left) and after release of stretch (right). Total force magnitudes are normalized by the initial baseline traction force magnitude, *F*_0_. Colors correspond to amount of applied stretch, with brighter colors corresponding to higher applied strain. (**c**,**d**) Plot of the change in force versus the change in cell length, ${\rm{\Delta }}x,$ for two states: the jump at onset of stretch, ${\rm{\Delta }}{F}_{st}$ (solid circles), and the drop at release of stretch, ${\rm{\Delta }}{F}_{un}$ (empty circles). Each set of the two conditions represents a single cell. Each change in force is relative to that cell’s baseline traction force magnitude before change in applied strain. High Pearson’s correlation coefficient values (*r*) suggest a strong linear relation. Linear best of fit lines (solid for ${\rm{\Delta }}{F}_{st}$ v. ${\rm{\Delta }}x,\,\,$and dashed for ${\rm{\Delta }}{F}_{un}$ v. ${\rm{\Delta }}x$) display a positive slope for both relations.

Our method allows the measurement of the cellular response to a step strain over timescales from a few seconds to a few hundred seconds. We measured the response of control cells to stretch and release for a range of applied strains between 1 and 20%, shown by the traces in Figure 2a,b. There, forces are normalized to the baseline traction force, *F*_0_, measured just before stretch, and strain magnitude is encoded by the color of the trace. Immediately after stretch, the traction forces were consistently above baseline levels, *i.e*. ${\rm{\Delta }}{F}_{st} > 0$. A few cells at the smallest applied strains showed a negative ${\rm{\Delta }}{F}_{st}$ (3/23 control and 0/26 vinculin KO) and were excluded from further analysis. After the application of stretch, the force remained elevated in most cells. Upon un-stretch, traction forces dropped below the original baseline. In nearly all cells, the force then increased toward the original baseline force, *F*_0_, plateauing at a new baseline, *F*_*f*_. Despite differences in baseline tractions, vinculin KO cells behaved qualitatively similarly to control cells during stretch and release (Fig. 2a,b).

The traces in Fig. 2a,b suggest that cells under larger deformations displayed larger changes in force. We tested how short-term changes in force are correlated to changes in cell length. We calculated the change in size of each cell upon the application of strain by multiplying the basal cell length by the applied strain, ${\rm{\Delta }}x\,=\,\varepsilon \,{L}_{cell}$. Over the range of applied strains, both the change in force upon stretch, ${\rm{\Delta }}{F}_{st}$_,_ and un-stretch, ${\rm{\Delta }}{F}_{un}$, increased linearly with ${\rm{\Delta }}x$, showing strong correlation in control cells (Fig. 2c; *r* = 0.70 for stretch, *r* = 0.77 for un-stretch) and moderate correlation in vinculin KO cells (Fig. 2d; *r* = 0.43 for stretch, *r* = 0.51 for un-stretch). Thus over short time-scales, the mechanical response for both cell types is linear.

### Stretched cells appear stiffer

**Figure 3:**
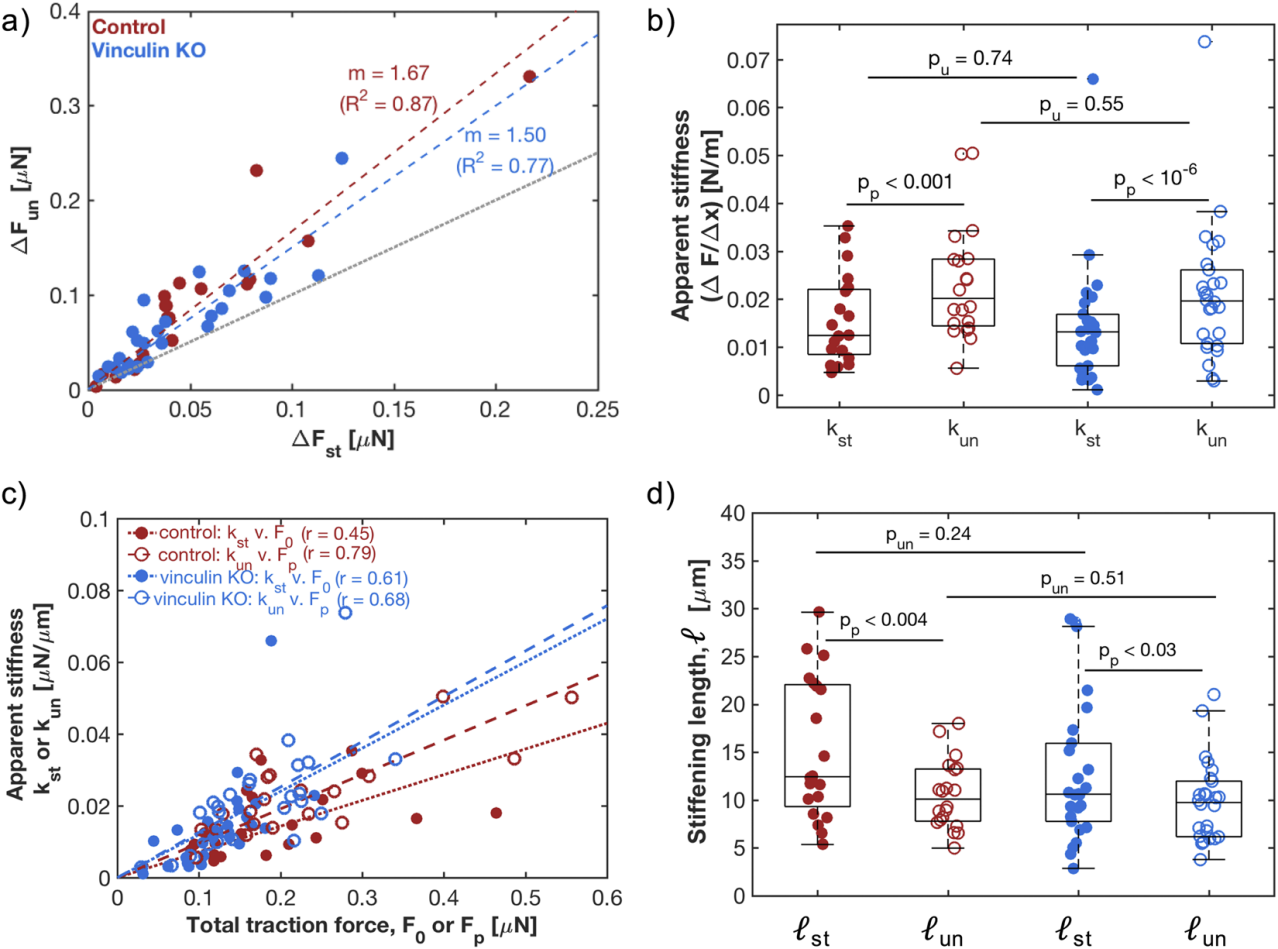
Stress-stiffening of stretched fibroblasts. (**a**) For each control cell (red) and vinculin KO cell (blue), ${\rm{\Delta }}{F}_{un}$ values plotted as a function of ${\rm{\Delta }}{F}_{st}$. Each data point represents a single cell, and the dashed line shows a linear best of fit through all data points, with fit slope *m*. ${R}^{2}$ represents the coefficient of determination for linear regression. The dotted gray line shows a slope of 1. (**b**) Apparent stiffnesses for control (red) and vinculin KO (blue) cells, at stretch, *k*_*st*_, and un-stretch, *k*_*un*_. Means from left to right: 0.016, 0.023, 0.014, and 0.021 N/m. (**c**) Stress stiffening shown through the apparent stiffness plotted as a function of total traction force before stretch (${k}_{st}\,v.\,{F}_{0}$, solid circles) and un-stretch (${k}_{un}\,v.\,{F}_{pl}$, empty circles). Moderate to high Pearson’s correlation coefficient values (*r*) suggest a linear relation. Dashed and dotted lines show the linear best of fit lines. (**d**) The stiffening length, $\ell ,$ for each individual cell in each condition, given as the ratio between total traction force and apparent stiffness. Means from left to right: 15.3, 10.5, 12.7, 9.7 µm. (**b**,**d**) Each data point represents a single cell, and the box and whisker plots summarize the entire population. The middle line represents the median of the population, while the bottom and top of the boxes represent the 1^st^ and 3^rd^ quartile, respectively. p-values were calculated by either a Welch’s t-test between conditions (p_u_) or a paired Student’s t-test within a condition (p_p_).

In a purely elastic system, the changes in force at stretch and at un-stretch would be equal. However, in the response of control cells, the force change at un-stretch, ${\rm{\Delta }}{F}_{un},$ was on average 67% higher than the force change at stretch, ${\rm{\Delta }}{F}_{st}$ (Fig. 3a red data; fit slope = 1.67, *R*^2^ = 0.87). Similarly, in vinculin KO cells, ${\rm{\Delta }}{F}_{un}$ was on average 50% higher than ${\rm{\Delta }}{F}_{st}$ (Fig. 3a blue data; fit slope = 1.50, *R*^2^ = 0.77). Since the application of strain is equal and opposite at stretch and unstretch, this suggests a stiffening of the cells in the stretched state.

Based on the linearity of the short-term responses to applied strains, we quantified apparent cellular stiffnesses using a conventional spring constant, $k={\rm{\Delta }}F/{\rm{\Delta }}x$ (Fig. 3b). We note some important points about this apparent cell ‘stiffness.’ First, it is a quantification of the response of the whole cell as a system, not as a material. In other words, we report a quantity analogous to a spring constant, not an elastic modulus. Second, this apparent stiffness may include contributions not only from conservative elastic forces and dissipative viscous forces, but also active forces generated by the cell. Thus, apparent stiffness quantifies the overall resistance of a cell to deformation, without differentiating between different sources of resistance.

By this measure, individual cells appeared to stiffen significantly during stretch (Fig. 3b). For control cells, the mean apparent stiffness increased from 0.016 N/m at stretch to 0.023 N/m at un-stretch (a > 40% increase, paired t-test; p = 2.5 × 10^−4^), while the mean of vinculin KO cells increased from 0.014 N/m to 0.021 N/m (a 50% increase, paired t-test; p = 1.4 × 10^−7^).

These observations of whole-cell apparent stiffness are reminiscent of the actin-dependent increases in cortical stiffness seen through MTC measurements of stretched cells^8^. A correlation between traction forces and cortical stiffness measurements has previously been reported^10,11^ and is proposed to originate from the contractile tension and non-linear rheology of the actin cytoskeleton^39^. In that spirit, we compared the apparent stiffness at stretch, *k*_*st*_, to initial traction forces just before stretch, *F*_0_. Additionally, we compared the apparent stiffness at un-stretch, *k*_*un*_, to plateau traction forces just before release of stretch, *F*_*pl*_ (Fig. 3c). Echoing cortical stiffness measurements, we found a significant correlation between apparent stiffness and the traction forces of the cell right before the change of state (*r* = 0.45–0.79).

A linear relationship between stiffness and force, as suggested by these data, would imply an exponential relationship between change in length, ${\rm{\Delta }}x,$ and force: $F({\rm{\Delta }}x)={F}_{o}{e}^{\Delta x/\ell }$. Here, the factor $\,\ell$, which we term the stiffening length, quantifies the onset of strain-stiffening. When $\Delta x\ll \ell$, the force response of the system varies linearly with strain (as in Fig. 2c,d), with a slope defined by the spring constant, $k={F}_{o}/\ell$. When ${\rm{\Delta }}x\gtrsim \ell$, the response is no longer linear, and strain-stiffening is significant. We calculated the stiffening length, $\ell ,$ for each cell as the ratio of baseline traction force to apparent stiffness (${\ell }_{st}={F}_{0}/{k}_{st}$ for stretch, and ${\ell }_{un}={F}_{pl}/{k}_{un}$ for un-stretch). In control cells, the stiffening length significantly decreased by 30% during stretch (from 15.3 to 10.5 *μ*m; p = 4.0 × 10^−3^; Fig. 3d). Similarly, the stiffening length decreased by 24% in vinculin KO cells (from 12.7 to 9.7 *μ*m; p = 0.03; Fig. 3d). Significant shortening of the stiffening length during stretch suggests that both cell types undergo changes to the cellular components that produce traction forces.

### Vinculin enhances mechanical irreversibility and energy dissipation

**Figure 4:**
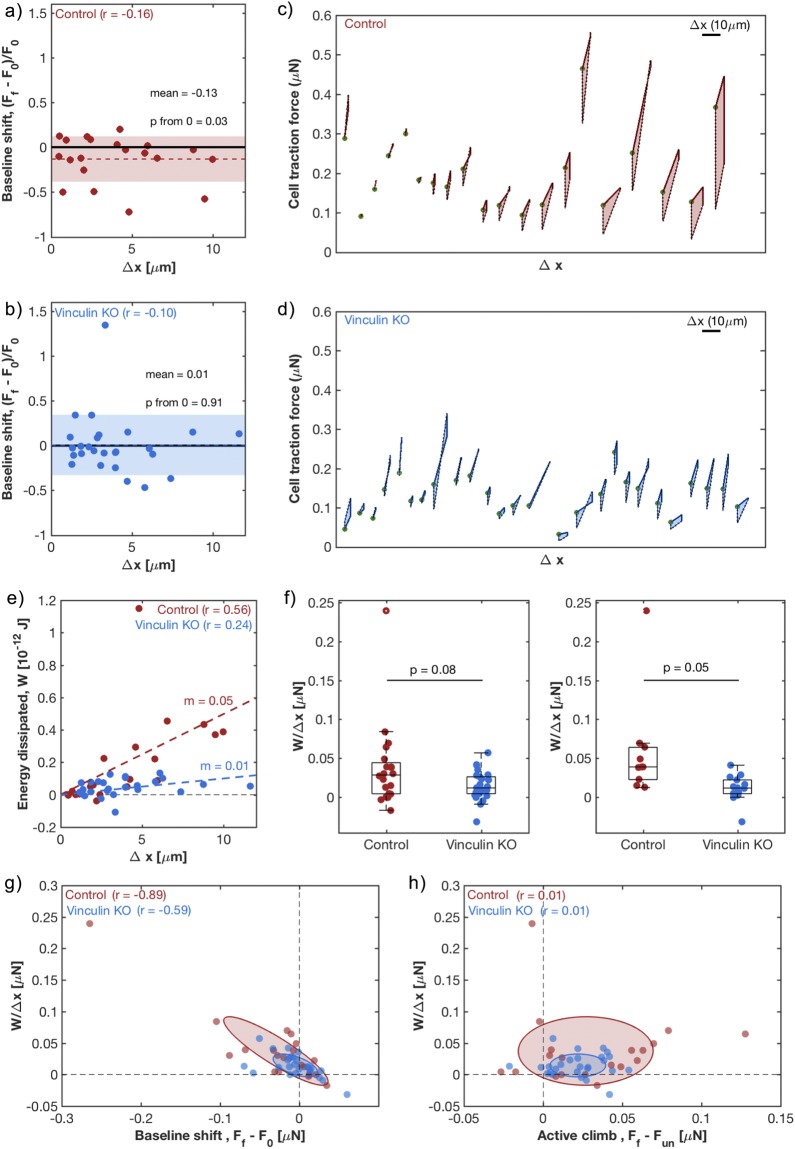
Dissipated energy in the stretch cycle is correlated with a baseline shift in traction forces. (**a**,**b**) Shift in baseline traction forces throughout the entire stretch and release process, ${F}_{f}-{F}_{0}$, normalized by the initial baseline, *F*_0_, for control (**a**) and vinculin KO (**b**) cells. Each data point represents a single cell and the colored region spans the mean +/− standard deviation. Low Pearson’s correlation coefficient (*r*) values suggest no relation between baseline shift and the change in cell length, ${\rm{\Delta }}x.$ (**c**,**d**) Force-displacement diagram for control (c) and vinculin KO (d) cells, showing total traction force of each individual cell throughout the cycle of stretch and release. Cells are arranged from left to right based on the area inside this curve, which is the dissipated mechanical energy. Scale bar shows the scale of ${\rm{\Delta }}x,$ change in cell length. Green dots indicate each cell’s initial traction force/zero displacement point. (**e**) Dissipated energy, *W*, for each cell as a function of cell length change. For control cells (red), a moderate *r* value and a positive fit slope (*m*) suggest a linear correlation. For vinculin KO cells (blue), a low *r* and small *m*, suggest a lack of correlation. (**f**) The dissipated energy per unit length, $\frac{W}{{\rm{\Delta }}x},$ across control (red) and vinculin KO (blue) cells, for the full population (left) and for cells with ${\rm{\Delta }}x.$>3 µm (right). Each data point represents a single cell while the box and whisker plots summarize the population. The middle line represents the median of the population, while the box top and bottom represent the 1^st^ and 3^rd^ quartile, respectively. p-values were calculated by Welch’s t-test. Means from left to right: 0.037, 0.014, 0.059, 0.011 µN. (**g**) Relationship between dissipation per unit length, $\frac{W}{{\rm{\Delta }}x},$ and baseline traction force shift,$\,{F}_{f}-{F}_{0}$. Moderate to high negative *r* values suggest a strong anti-correlation. (**h**) The relationship between the dissipation per unit length, $\frac{W}{{\rm{\Delta }}x},$ and active climb in force after un-stretch,$\,{F}_{f}-{F}_{un}$. Very low *r* values suggest a lack of relation. (**g**,**h**) Each data point represents a single cell. The axes of the ellipses are the eigenvectors of the covariance matrix between *x* and *y* coordinates. The widths of the ellipses are the square root of the variances along these vectors, showing the variation in the populations.

To further probe the mechanical changes in cells in response to a cyclic stretch, we compared the final force ten minutes after release of stretch, *F*_*f*_, to the initial baseline force before stretch, *F*_0_. On average, the final traction force in control cells showed a small but significant reduction (mean 13% below its initial value; p = 0.03; Fig. 4a), while vinculin KO cells returned, on average, to initial traction force levels (mean 1% above the baseline value; p = 0.91; Fig. 4b). However, both populations showed a high degree of variability. Final traction forces (normalized by their baseline values) had a standard deviation of 26% for control and 34% for vinculin KO, respectively. In both populations, this baseline shift showed no correlation with the amount that the cell was stretched (Fig. 4a,b).

To quantify mechanical changes throughout the entire process, we measured the mechanical energy dissipated by the cell during the cycle, *W*. The dissipated energy is simply calculated as the area enclosed by the path of the cell on a force-displacement diagram (Fig. 4c,d). It measures the difference between mechanical energy put into the cell during stretch and mechanical energy returned to the ECM upon release. For a purely elastic object, this quantity is zero. It is positive when the object is visco-elastic or plastic, with internal friction or bond-breaking. In these panels, each enclosed path represents a cell, and the cells are sorted from left to right based on the dissipated energy.

Apparently, the dissipated energy in the control cells is larger than the dissipated energy in the vinculin KO cells. In control cells, the dissipated energy, *W*, shows a strong correlation to the change in cell length, ${\rm{\Delta }}x$ (r = 0.56 and fit slope = 0.05; Fig. 4e). In contrast, vinculin KO cells have little correlation, with low amounts of dissipated energy regardless of the applied strain (r = 0.24 and fit slope = 0.01; Fig. 4e).

We compared the dissipation normalized by the change in cell length, $\frac{W}{{\rm{\Delta }}x},$ across the two populations (Fig. 4f). When considering the full range of deformations, the populations are quite similar (mean for control = 0.04 µN, mean for vinculin KO = 0.01 µN; p = 0.08 by t-test, p = 0.10 by Kolmogorov-Smirnov test; Fig. 4f, left). However, when considering only larger deformations, ${\rm{\Delta }}x$> 3 µm, control cells show a larger, more significant increase in dissipated energy (the mean for control = 0.06 µN, mean for vinculin KO = 0.01 µN; p = 0.05 by t-test, p = 0.01 by Kolmogorov-Smirnov test; Fig. 4f, right).

As might be expected for a system undergoing plastic deformation, mechanical dissipation is correlated to changes in baseline traction forces: the normalized dissipation shows a strong negative correlation with the baseline shift, F_f_ − F_0_ (r = −0.89 for control cells, r = −0.59 for vinculin KO cells; Fig. 4g). On the other hand, we see no correlation between the normalized dissipation and the magnitude of the recovery after the release of the stretch, F_f_ − F_un_ (r = 0.01 for control, r = 0.01 for vinculin KO; Fig. 4h). This suggests that the irreversibility in the baseline forces is not driven by a suppression of cell contractility, but by dissipative passive processes, such as plastic deformation or slip.

## Conclusion

We have measured changes in traction forces of adherent cells throughout stretch- and release of the ECM. Our experiments show an increase of apparent stiffness in response to deformation and stress, similar to previous reports of reconstituted biopolymer networks^40,41^ and live cells^8,10,11,39,42–44^. Under higher applied strains, cells expressing vinculin not only stiffen more, but they also experience a greater dissipation of mechanical energy, which is correlated with a loss of mechanical reversibility.

Vinculin has known roles in cell contractility^23,24^, adhesion and spreading^23,25–27^. Its place in established mechano-sensing pathways involves interactions with actin and other focal adhesion proteins^31^. Our observation that vinculin increases baseline forces is consistent with these previous findings. On the other hand, we did not observe a vinculin dependent change in apparent stiffness (Fig. 3b, unpaired t-test; p = 0.74 for *k*_*st*_ and p = 0.55 for *k*_*un*_). This seems inconsistent with previous reports of vinculin-dependent stiffening of the cortical cytoskeleton^23,26,45–47^. We see two possible reasons for this discrepancy. First, as shown in this work and in previous reports^10,11^, cell stiffness increases with baseline traction forces. While our vinculin KO cells showed a 36% drop in baseline traction stresses, previous studies found a much larger decrease (e.g. 60% in^23^). Second, cortical cytoskeletal stiffness may not be the only significant contributor to the apparent stiffness measured here. It may also be impacted by active contractility generated in opposition to applied force, and by other passive contributions from the cell (*e.g*. the stiffness of non-cortical actin, non-actin cytoskeletal proteins, adhesion sites, and the nucleus). Further studies should assess the relative contributions of these factors in the apparent stiffnesses measured with this method.

The reorganization of the cytoskeleton in response to stretch has previously been interpreted as fluidization^18–20,43^. This was inferred from a drop in traction forces after a transient stretch-and release cycle, which is consistent with our observations. However, armed with additional information on the traction forces during the stretched state, we find that the cells actually increase traction stresses and increase their apparent stiffness in the stretched state. The drop in traction forces upon un-stretch is therefore attributed not to fluidization, but to limited mechanical plasticity as suggested by recent experiments with magnetic tweezers^48^. Future experiments should test this idea by repeated stretching of cells, with repetition rate as a key parameter.

In both of our cell types, dissipation of mechanical energy is strongly correlated with a drop in baseline traction forces, but not with the slow recovery of traction forces after release. This further suggests that the lack of mechanical reversibility originates from plastic deformation in the cytoskeleton or slip at the focal adhesions, rather than changes in active contractility. High resolution imaging of actin and focal adhesion organization and localization could help reveal such changes.

To our knowledge, the dissipation of mechanical energy in a cell during a mechanical cycle has not been previously reported. Consistent with the conventional role of vinculin as a mechanotransducer, enhanced dissipation in the presence of vinculin is likely due to down-stream remodeling of the cytoskeleton. For example, vinculin may play a role in regulating stress fiber formation through myosin and Rho. Recent reports of optogenetically-induced cell contraction show a Rho-dependent dynamic recruitment of myosin and actin within minutes^49,50^. Evidence also suggests that vinculin may play a role in the regulation of the branched actin network of the cell, since it can bind to the Arp2/3 complex^51^. Further investigations should explore the cell’s response to deformation on both shorter and longer timescales in order to further illuminate the mechanisms that control this response.

Cells need to recover from mechanical deformations, and adapt to changing environments. By stiffening in response to applied strains, cells become less susceptible to further deformations. Our results suggest that vinculin enhances this adaption, through stretched-induced remodeling. Further experiments are required to identify the mechanisms of remodeling and its dependence on other physical stimuli, including the stiffness of the ECM and the time-scale of deformation.

## Electronic supplementary material

Supplementary Figures

## Data Availability

The datasets generated during and/or analysed during the current study are available from the corresponding author on reasonable request.
