# Supplementary material for: Vinculin and the mechanical response of adherent fibroblasts to matrix deformation
Source: Sci Rep. 2018 Dec 19;8:17967. doi: 10.1038/s41598-018-36272-9 (PMC6299284; doi:10.1038/s41598-018-36272-9)
Supplement: Supplementary file 1 — Supplementary Figures [file 41598_2018_36272_MOESM1_ESM.pdf]

Vinculin and the mechanical response of adherent fibroblasts to matrix deformation

Kathryn A. Rosowski[1,2†], Rostislav Boltyanskiy[2†], Yingjie Xiang[2], Koen Van den Dries[3], Martin A. Schwartz[3,4], and Eric R. Dufresne[1,2*]

1 Department of Materials, ETH Zürich, 8093, Zürich, Switzerland
2 Department of Mechanical Engineering and Materials Science, Yale University, New Haven, CT, 06511, USA
3 Cardiovascular Research Center and Department of Medicine (Cardiology), Yale University School of Medicine, New Haven, CT, 06511, USA
4 Departments of Cell Biology and Biomedical Engineering, Yale University, New Haven, CT, 06511, USA

†these authors contributed equally
*corresponding author: eric.dufresne@mat.ethz.ch

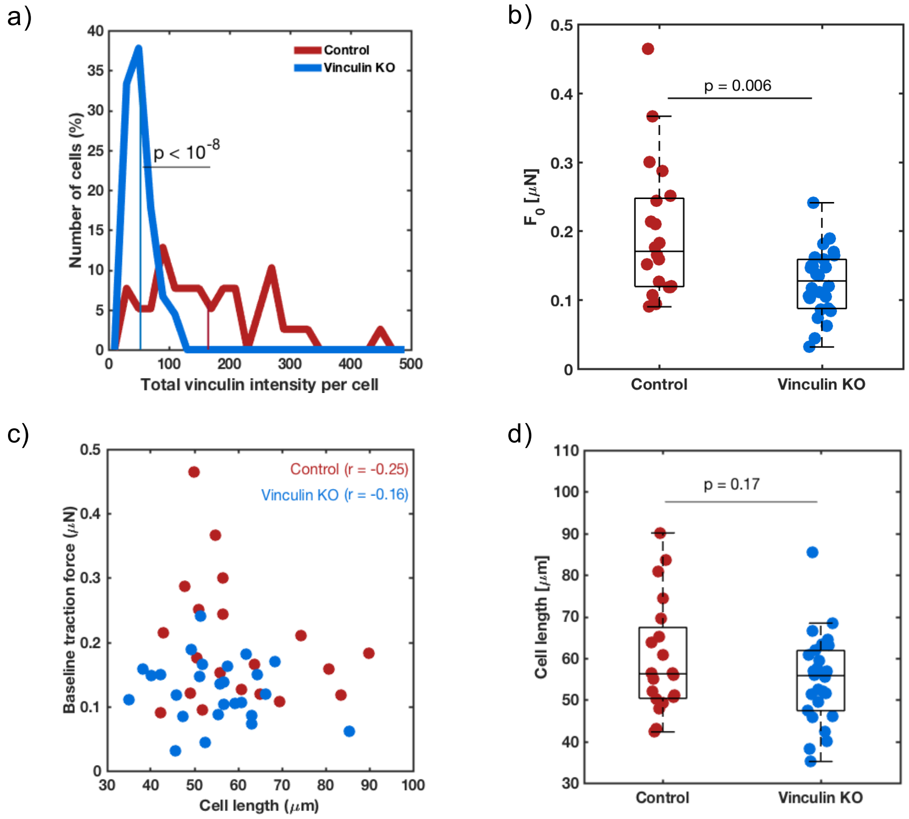

Supplementary Fig S1. Loss of vinculin in KO cells leads to lower baseline traction forces

a) Histogram of vinculin fluorescent intensity integrated over the whole cell, for control (red) and vinculin KO (blue) cells. Vertical lines show the means for the populations, which are significantly different. b) Initial baseline traction forces, F_0, for control (red) and vinculin KO (blue) cells. Means from left to right: 0.197, 0.126 μN. c) Scatter plot of cell length vs. the baseline traction force of each cell. Low Pearson's correlation coefficient values (*r*) suggest a lack of correlation for each popoulation. d) Cell length measurements for the two populations of control (red) and vinculin KO (blue) cells. b,d) Each data point represents a single cell, and the box and whisker plots summarize the entire population. The middle line represents the median of the population, while the bottom and top of the boxes represent the 1st and 3rd quartile, respectively. a,b,d) p-values were calculated by a Welch's t-test between conditions.

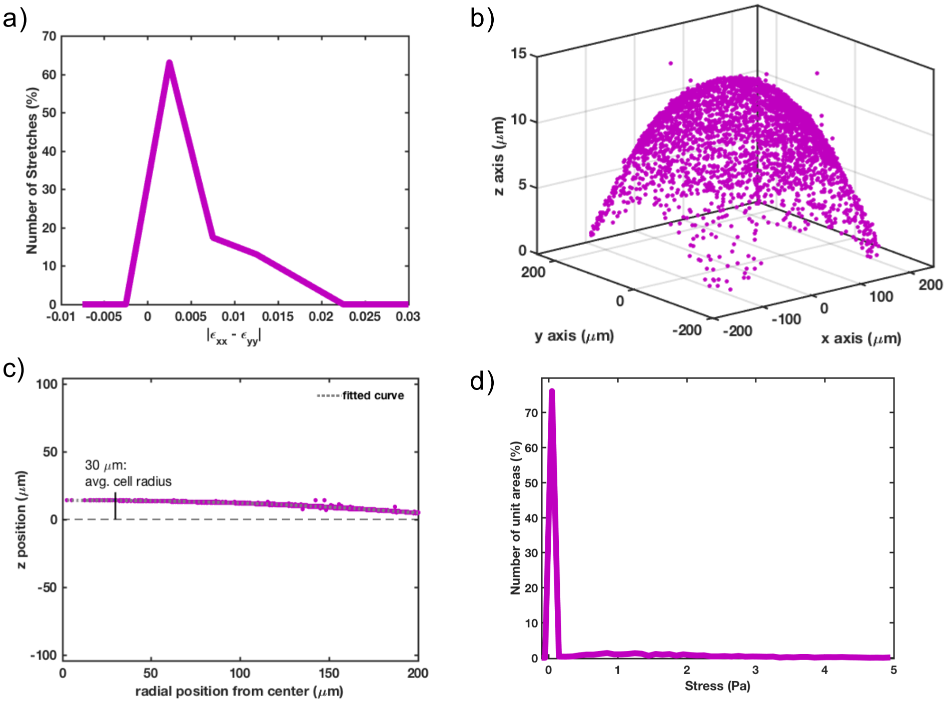

Supplementary Fig S2. Characterization of the stretch induced by indentation

a) A histogram showing the degree to which the applied strain is biaxial. The difference between strain in x (ϵ_{xx}) and strain in y (ϵ_{yy}) is small for all stretched cells. Control and vinculin KO experiments are shown here pooled together. b) 3D scatter plot of fluorescent beads on the silicone substrate surface, tracked after an indentation of about 400 µm (giving a 13% strain in the middle of the indentation). c) Radial collapse of the beads in (b), showing the z-displacement of the substrate surface due to indentation. This surface is fit to a parabola (dotted line). A black line shows that at half the length of a typical cell, centered in the middle of the indentation, the z-displacement is minimal. d) Measurement of the error in affine calculations of applied strain. The strain of a substrate with no cells was calculated as an affine transformation. This transformation was applied to the bead positions in the unstretched state, and then the beads were tracked between these positions and the true stretched-state positions. From these displacements, stresses were calculated and plotted up in a histogram to show the possible contribution to traction force calculations due to errors in strain calculations.
